# Searching for plasticity in dissociated cortical cultures on multi-electrode arrays

**DOI:** 10.1186/1477-5751-5-16

**Published:** 2006-10-26

**Authors:** Daniel A Wagenaar, Jerome Pine, Steve M Potter

**Affiliations:** 1 Department of Physics, California Institute of Technology, Caltech 103-33, Pasadena, CA 91125, USA; 2 Present address: Division of Biological Sciences, Neuroscience Section, University of California at San Diego, 9500 Gilman Drive, La Jolla, CA 92093, USA; 3 Department of Physics, California Institute of Technology, Caltech 256-48, Pasadena, CA 91125, USA; 4 Coulter Department of Biomedical Engineering, Georgia Institute of Technology and Emory University, 313 Ferst Drive, Atlanta, GA 30332-0535, USA

## Abstract

We attempted to induce functional plasticity in dense cultures of cortical cells using stimulation through extracellular electrodes embedded in the culture dish substrate (multi-electrode arrays, or MEAs). We looked for plasticity expressed in changes in spontaneous burst patterns, and in array-wide response patterns to electrical stimuli, following several induction protocols related to those used in the literature, as well as some novel ones. Experiments were performed with spontaneous culture-wide bursting suppressed by either distributed electrical stimulation or by elevated extracellular magnesium concentrations as well as with spontaneous bursting untreated. Changes concomitant with induction were no larger in magnitude than changes that occurred spontaneously, except in one novel protocol in which spontaneous bursts were quieted using distributed electrical stimulation.

## Background

Cultured neuronal networks can be used as models for the study of the cellular and network properties that underlie learning, memory, and information processing [1-5]. Cultures of dissociated neurons and glia on multi-electrode arrays (MEAs) are a very attractive model system for studying both structural and functional plasticity, since they make it possible to record from the same set of neurons for several months [6-8] – as opposed to mere hours for intracellular experiments. Furthermore, it is much easier to image a network in culture over time [9] than it is to image an intact brain at the cellular level [10]. By considering electrical stimuli delivered by MEA electrodes as artificial sensory input, and recorded signals as analogous to motor outputs, one can make in vitro studies more relevant to in vivo neural processing. By closing the sensory-motor loop around a culture, for example, by connecting it to an artificial [11] or robotic [12,13] embodiment, neural plasticity in vitro can serve as a simpler and more accessible model for learning and memory studies than intact lab animals.

An essential component of the implementation of learning and memory in vertebrates is changes to the connections between cortical neurons. Such changes can take the form of the extension or retraction of neurites and spines, accompanied by the formation or elimination of synapses, or they can take the form of strengthening or weakening of existing synapses (e.g. [14-16]). In culture, plasticity in individual synapses can be induced by forcing the postsynaptic cell to fire either just before or just after the synapse has been activated using intracellular electrophysiology [17,18]. By cleverly manipulating visual inputs, Fu *et al.*[19] have shown that such 'spike timing dependent plasticity' (STDP) can also be made to occur in the cortex *in vivo*. Indeed, STDP appears to occur throughout the brain; see [20] for a recent review of results both *in vivo *and in slice.

While changes in the anatomical and functional connectivity in neural tissue take place on time-scales from milliseconds to years, changes that occur rapidly yet stay in effect for a long time are particularly interesting because of their relevance to memory formation. This is why we, as well as may other researchers, focus on them. Accordingly, for the purpose of this report, we define functional plasticity as those changes in stimulus – response relationships or in spontaneous activity patterns, that are experimentally induced by electrical stimulation, and lasting at least on the order of one hour. Thus, long-term potentiation (LTP) [21] and long-term depression (LTD) [22,23] would be included in the definition, but paired pulse facilitation and depression would not, nor would spontaneously occurring changes or developmental changes.

The history of published MEA studies demonstrating functional plasticity in cultured networks began in the 1990s. The research group of Akio Kawana at NTT in Japan reported that tetanic stimulation through one or several electrodes resulted in plasticity [24]. They observed a change in the probability of evoking bursts by test pulses, as well as a change in the rate of spontaneous bursting, as a result of repeatedly evoking bursts using strong tetanic stimulation. Jimbo et al. observed similar results with more modest tetani, and used voltage clamp to observe inward currents associated with evoked bursts [25]. After tetanization, the onset latencies of these currents were earlier and more precise. The following year, Jimbo et al. reported that tetanizing a single electrode resulted in changes in the responses to test pulses to other electrodes [26]. Culture-wide responses to a particular stimulation electrode were either all upregulated or all downregulated, a phenomenon they called 'pathway-dependent plasticity'. Individual pathways (defined as responses throughout the array to stimuli on one particular electrode) were upregulated or downregulated depending on the correlation between (pretetanus) responses to stimuli applied to the test electrode and to the tetanization electrode. In a final paper, simultaneous tetanization through a pair of electrodes was used to induce more subtle forms of plasticity, expressed in detailed spike patterns evoked by electrical (probe) pulses [27].

Since then, a few other groups have reported on other forms of plasticity in MEA neural cultures. Typically, these later papers have focused on more abstract plasticity results, seemingly requiring network-level interpretations rather than synapse-level ones. For instance, Shahaf and Marom reported that networks could be made to learn to respond in specific ways to test pulses, by repeatedly stimulating until the desired response was obtained [3], while Ruaro et al. reported that cultured networks could learn to "extract a specific pattern from a complex image" that had been presented repeatedly as spatial patterns of multielectrode stimulation [5].

An overview of the protocols and principal results of each of the above-mentioned papers is given in Table 1. To the best of our knowledge, no peer-reviewed reports by other research groups verifying any of these results have been published to date. As a result, whether cortical cultures can, in fact, learn is currently a subject of controversy [28]. At least, it appears that the conditions in which plasticity can be induced in dissociated cortical cultures using extracellular electrical stimuli are subtle and not very well understood.

**Table 1:** Overview of plasticity-inducing stimuli used by other researchers. The following is a very brief synopsis of the methods and main results of a number of previous studies that reported plasticity in dense cortical cultures on MEAs. Please refer to the original papers for more information.

**Ref.**	**Induction stimuli**	**Test stimuli**	**Results**
Maeda et al. (1998) [24]	Trains of 20 pulses at 20 Hz simultaneously to each of 5 electrodes, repeated 5–10× at 10–15 s intervals.	Trains of 20–30 pulses at 1 kHz or stronger single pulses, to 1 or 5 electrodes, repeated every 15–30 s.	Increased probability of evoking array-wide bursts by test stimuli after tetanization.
Jimbo et al. (1998) [25]	Trains of 11 pulses at 20 Hz to a single electrode, repeated 10× at 5 s intervals.	Single pulses, repeated every 10 s.	As above, plus earlier and more precisely timed onset for intracellular inward currents due to evoked bursts.
Jimbo et al. (1999) [26]	Trains of 10 pulses at 20 Hz to one electrode, repeated 20× at 5 s intervals.	Individual pulses to each of 64 electrodes, repeated 10× at 3 s intervals.	'Pathway- dependent' plasticity.
Tateno and Jimbo (1999) [27]	As above, as well as simultaneous tetanization of a pair of electrodes.	Individual pulses to the tetanized electrodes, repeated 53×.	Increased response to test pulses after paired tetani, with improved temporal precision of first response spikes.
Shahaf and Marom (2001) [3]	Bipolar stimulation between a pair of electrodes, at 1–3 s intervals, repeated until the desired response was seen, or for 10 min max.	*Induction stimuli served as test stimuli*.	Desired responses (increased spike rate 50–60 ms post-stimulus) obtained after fewer trials on successive test series.
Ruaro et al. (2005) [5]	Trains of 100 pulses at 250 Hz simultaneously to each of 15 electrodes in an L-shape, repeated 40× at 2 s intervals.	Stimuli, simultaneously to several electrodes, in an L- or ⌉-shape.	Responses to L-shape enhanced relative to ⌉-shape.

As a necessary prerequisite to studying learning and memory in MEA cultures, we sought to demonstrate reliable functional plasticity using extracellular stimulation protocols similar to some of those mentioned above. One protocol, in which bursting was quieted with distributed multi-site stimuli [29] showed a small but statistically significant plasticity, but all other protocols failed to show functional plasticity (in the sense defined above). We discuss the implications of effects of spontaneous population bursting on plasticity in cultured networks.

## Results

### Confirmation of cultures' basic physiological properties

Since we describe mostly negative results, it was critical to make sure that positive results *could *have been obtained. That is, the stimulation and recording systems must be working, the preparations healthy, and their spontaneous activity and responses to test pulses comparable to those observed in cultures in which induced plasticity has been reported by others. Similarity in reaction to common pharmacological agents should also be confirmed.

Our cultures passed each of these checks:

Spontaneous activity

The spontaneous activity of our cultures consisted of interspersed firing of several cells at low rates, interrupted by culture-wide bursts at varying intervals [30]. This is similar to the behavior of the cultures used by the NTT group [31] and others [32,8].

Responses to test pulses

As reported before [33], we observed individual spikes and short trains of spikes on many electrodes in response to electrical stimulation on a single electrode, just as the NTT group did [26]. In addition, culture-wide bursts were observed in response to some stimuli, in agreement with the findings of [24].

Reactions to pharmacological manipulations

**Figure 1:**
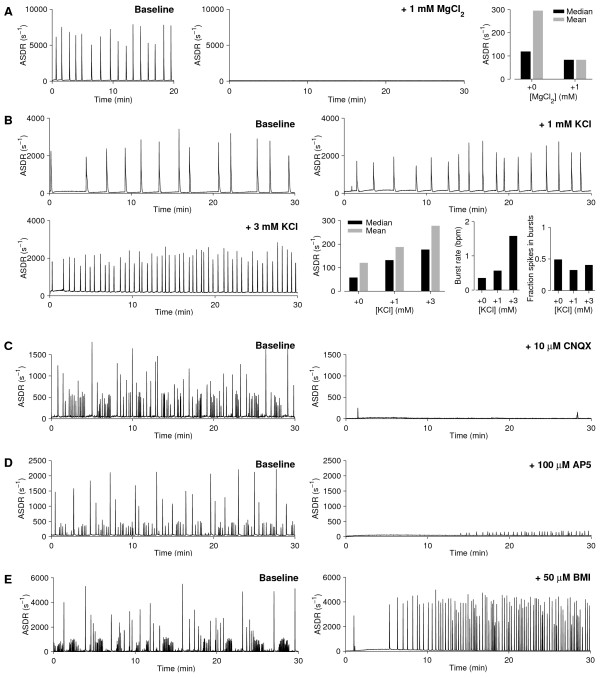
**Reactions to pharmacological manipulations**. **A **Adding 1 mM Mg^2+ ^(to the baseline of 0.8 mM) stopped spontaneous bursts, and reduced the array-wide spike detection rate (ASDR) outside of bursts slightly. **B **Adding 1 or 3 mM K^+ ^(to the baseline of 5.4 mM) increased burst rates and inter-burst firing rates. The fraction of spikes that occurred inside bursts (as opposed to between bursts) remained similar. **C** CNQX, an AMPA channel blocker, inhibited bursting and reduced baseline ASDR. **D **AP5, an NMDA channel blocker, inhibited bursting for a limited period of time. **E **Bicuculline methiodide (BMI), a GABA channel blocker, increased burstiness. (Data for *A–E *were obtained from different cultures, *N *= 1 for each substance. Baselines were recorded immediately prior to adding drugs. Since the results were fully consistent with expectations, a more in-depth investigation was deemed unnecessary.)

An increased magnesium concentration in the medium reduced or abolished burstiness, presumably by blocking the calcium channels of NMDA receptors (Figure 1A). An increase in burst frequencies and inter-burst spike rates was obtained by adding potassium (Figure 1B), presumably through shifting the resting membrane potential: adding 3 mM K^+ ^(to the baseline of 5.8 mM) should result in a depolarization by about 11 mV. With NMDA receptors blocked by AP5 (100 *μ*M), bursting ceased (Figure 1C). Blocking AMPA receptors with CNQX (10 *μ*M) also prevented bursting, and reduced inter-burst spike rates (Figure 1D). Conversely, bicuculline, a blocker of GABA receptors, increased burst rates at a concentration of 50 *μ*M (Figure 1E).

**Figure 2:**
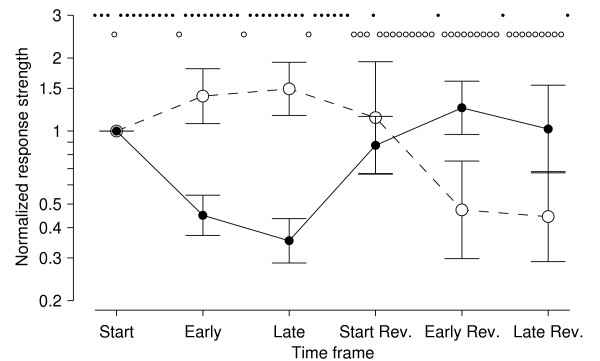
**Confirmation of the elasticity results of Eytan et al. (2003) [34]**. One electrode was initially stimulated at 1 Hz for one hour (*solid symbols*), while another was stimulated at 1/60 Hz (*open symbols*). Then, the roles were reversed. The graph shows the number of spikes recorded array-wide, 15–30 ms after a stimulus, normalized to the value at the beginning of the experiment. 'Start' refers to the first stimulus to the 'slow' electrode, or the average of the first 5 stimuli to the 'fast' electrode; 'Early' refers to the average of the first 5 stimuli to the 'slow' electrode, or the average of the 5 × 4 surrounding stimuli to the 'fast' electrode; 'Late' refers to average of the last 20 stimuli to the 'slow' electrode, or the average of the last 1200 stimuli to the 'fast' electrode. (This slightly unusual way of organizing the data was used to balance the need to collect sufficient statistics with the desire to measure as close as possible to the beginning of the experiment.) Data are mean ± SEM (in log-space) from 16 experiments on 4 cultures. The sequence of open and closed symbols near the top of the graph are a cartoon of the stimulation sequence; the actual number of stimuli was much greater.

We also tested whether our cultures exhibited the 'elastic' changes in response strength observed in [34]. They found that when two electrodes were repeatedly stimulated, one at a very slow rate (0.02 Hz) and one at a faster rate (0.2 Hz), the responses to the 'slow' electrode were enhanced while the responses to the 'fast' electrode are weakened, effects which were fully reversible. In our tests, we stimulated one electrode, *A*, at 1 Hz for one hour, while stimulating another, *B*, at 1/60 Hz. Indeed, responses to electrode *A *decreased significantly (*p *< 0.001; *N *= 16 electrode pairs in 4 cultures), while responses to electrode *B *appeared to increase slightly (*p *= 0.06; Figure 2). Then, the roles were reversed for one hour – *B *was stimulated at 1 Hz, and *A *at 1/60 Hz – and soon responses to *A *increased back to baseline or perhaps slightly above (*p *= 0.2), while responses to *B *decreased significantly (*p *< 0.05), in agreement with [34]. In conclusion, our cultures are healthy, and – by all measures we tested – are similar to those used by other researchers.

### Overview of protocols

We looked for plasticity induced by electrical stimulation in three series of investigations: *Changes induced in burst patterns, Changes in stimulus-response maps*, and *Changes in specific responses*. Within each series, we performed experiments with several different protocols. Before describing the methods and results in detail, we provide in this section an overview of our protocols.

Series I: Changes induced in burst patterns

If a plasticity-inducing stimulus sequence has an effect on many synapses, it should have an effect on a culture's overall activity, and in particular on its spontaneous culture-wide bursts. Strong stimuli, delivered through several electrodes in parallel, should have the best chance of inducing such global plasticity. To test this hypothesis, we recorded spontaneous activity before and after attempting to induce plasticity using strong stimuli, and measured burst frequencies, sizes, and the total number of spikes in bursts per unit time. In similar experiments, [24] found that burst frequencies increased following tetanization.

Series II: Changes in stimulus – response maps

According to [26], tetanization through a single electrode can induce changes that are stimulation-site specific, that is, array-wide responses to test stimuli on a given electrode (not necessarily the tetanized electrode) are either all upregulated or all downregulated. To test this hypothesis, we recorded responses to test pulses delivered sequentially to each electrode in the array before and after tetanization. Then we asked two questions: (1) Is there any change in how strongly individual recording sites respond to particular stimuli? (2) Are such changes stimulation-site specific (as reported by [26]), recording-site specific, or more complexly distributed?

Series III: Changes in specific responses

From intracellular recording experiments, it is well known that tetanizing a pair of cells can strengthen or weaken synapses between those cells depending on the timing of the tetanizing stimuli. MEA electrodes do not provide direct access to pairs of cells with known synaptic connectivity, but if one electrode records responses both after stimulation to electrode *A *and to electrode *B*, it is likely that shared synaptic pathways exist. Therefore, tetanizing the pair *A *and *B *can be expected to affect the responses on the shared target. To test this hypothesis, we selected pairs of stimulation electrodes that both evoked responses at a third site, recorded those responses, and compared them before and after paired-pulse tetanization.

These protocols were chosen because of their relative simplicity, and because their expected results have an intuitive connection to established properties of LTP and LTD induction in individual pairs of cells (compare [24,26] and [18]). We hoped that this would make it easier to obtain positive results. Viewed in this light, the more abstracted learning described by [3] or [5] would be less obvious starting points for studying the generalizability of plasticity results. (Note that our choices were in no way politically motivated, nor do we intend to cast doubt on any specific results previously reported.)

In all experiments, spontaneous or test-pulse-evoked activity was recorded for two hours (or more) before and two hours after the induction sequence. The activity in the first hour after induction (the "post" period) was then compared to the activity in the last hour before (the "baseline" period), to determine the changes associated with the induction sequence. Importantly, the activity in the hour before induction was also compared to the activity one hour before that (the "control"), to estimate the magnitude of spontaneous changes attributable merely to drift or random variability. This is critical, because drift typically substantially exceeds inter-trial variability in recordings from dissociated cultures on MEAs. Statistical tests were applied to determine whether changes concomitant with the induction sequence were larger than spontaneous changes. Each protocol was tested on multiple cultures. These experiments should have had enough statistical power to discover plastic changes if any of the effects previously reported occurred in our cultures.

### Multiple ways of handling culture-wide bursts

A large part of the spontaneous activity of dense cortical cultures on MEAs consists of globally synchronized intense bursts [31,32,8,30]. These bursts often contain thousands of spikes in a brief period (0.1–2 s), and should be distinguished from bursts consisting of only a few spikes recorded from individual cells. We previously hypothesized that this ongoing spontaneous bursting activity may interfere with inducing plasticity and maintaining changes [29]. Therefore, in addition to experiments under baseline conditions, we used two different methods to reduce bursting. One was to add 1 or 2 mM magnesium chloride to the medium (baseline concentration of Mg^2+^: 0.8 mM). This transiently reduced or abolished spontaneous bursting, presumably by reducing NMDA channel conductance (see Figure 1). Note that even though partially blocking NMDA channels could be expected to affect LTP and LTD, this same method of reducing bursting was used in [24], apparently without negatively affecting plasticity. The other method we used was distributed electrical stimulation [29], which completely suppressed bursting for as long as it was applied. Distributed electrical stimulation, when used, was also applied for the entire duration of the experiment, so that any potential (unintentional) short-term or long-term plasticity it might cause would not confound our tests for plasticity caused by the (intentional) induction protocols. (Note that in previous work [29] we saw no plastic effects from burst quieting.)

We shall now proceed to describe each of the three series of experiments in detail.

### Series I: Changes induced in burst patterns

We tested whether strong stimuli could induce changes in spontaneous bursting behavior in 10 cultures. We measured the number of bursts spanning at least 10 electrodes in one-hour windows before and after an induction sequence, as well as the number of spikes in those bursts. Very strong stimuli were used as induction sequences in these experiments. In most cases, several experiments were performed consecutively on one culture, with several hours between experiments.

#### Details of induction sequences

Induction consisted of volleys of pulses to 5–10 electrodes. Electrodes were chosen on the basis that they evoked strong responses when stimulated individually (see *Choice of electrodes*, under *Methods*). Within a volley, each electrode received one pulse, and successive electrodes were stimulated at 2–5 ms intervals (inter-electrode interval; IEI). Such volleys had a high probability of evoking bursts, which, according to [24], is essential for affecting later spontaneous bursting. Volleys were either delivered singly, or in sets of 4 or 20 with an inter-volley interval (IVI) of 50–500 ms. A pause of 5–10 s was interposed between sets, so that each set had a good chance of evoking bursts. (In general, evoking bursts was subject to a relative refractory period on the order of 1 s [31].) The full induction sequence lasted 8–17 min. The precise protocols used in this series are listed in Table 2.

**Table 2:** Details of experiments on plasticity expressed in burst patterns (Series I).

**Protocol**	**Tetanus**	**Conditions**	**No. and ages of cultures**	**Total expts.**	**Intervals**^*a*^
**I.1**	Sets of 4 volleys (IVI: 500 ms) to 10 geometrically close electrodes (IEI: 5 ms), repeated every 5 s for 15 min.	Baseline medium, spontaneous bursting.	2 × 2 ^*b*^; 10–19 div	4	-
**I.2**	Single volleys to 5 electrodes (IEI: 2 ms), repeated every 10 s for 17 min.	Baseline medium, spontaneous bursting.	4; 13–16 div	16	4 h
**I.3a**	Single volleys to 8 electrodes in a vertical column (IEI: 2 ms), repeated every 10 s for 15 min.	Elevated magnesium (1–2 mM) to reduce spontaneous bursting.	3; 18–20 div	6	2 h
**I.3b**	Sets of 20 volleys (IVI: 50 ms) to 8 electrodes in a vertical column (IEI: 2 ms), repeated every 5 s for 8 min.	Elevated magnesium (1–2 mM) to reduce spontaneous bursting.	1; 17 div	2	2 h

^*a *^Between experiments on a single culture.

^*b *^Two cultures were each used twice, 6 days apart, resulting – for practical purposes – in four independent experiments.

#### Data analysis and results

To test whether stimuli had an effect on spontaneous bursting, we counted the number of bursts in the hour immediately before the induction sequence (*N*_base_), as well as in the hour after (*N*_post_). In order to be able to test whether the change concomitant with the induction sequence was larger than changes that occurred spontaneously, we also counted bursts in the hour before the baseline hour, called the control hour (*N*_ctrl_). We then computed the absolute value of the change concomitant with the induction sequence, Δ*N*_ind _= |*N*_post _- *N*_base_|, as well as the spontaneous change, i.e., the change attributable to drift, Δ*N*_spont _|*N*_base _- *N*_ctrl_|.

**Figure 3:**
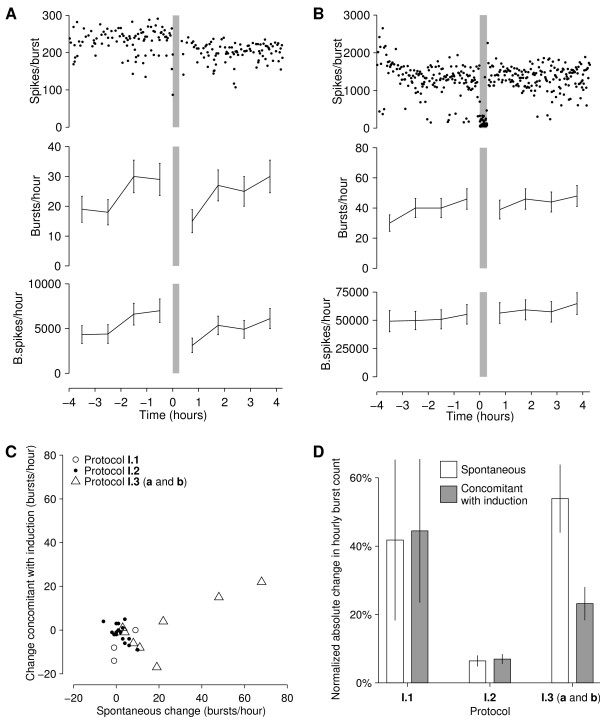
**Results of Series I: Changes induced in spontaneous bursting by strong stimulation through several electrodes**. **A **An exceptional example from protocol **I.1**, where the induction sequence resulted in reduced burst rates and sizes. Note though, that spontaneous drift in the burst rate before the analyzed portion of the recording was of comparable magnitude. **B **A typical example from protocol **I.2**, showing no effect. Induction sequences in *A *and *B *are marked by gray bars. Top to bottom: number of spikes in individual bursts; number of bursts in successive one-hour time windows (with error bars based on assumed Poisson statistics); total number of spikes in bursts in successive hours. **C **A summary of all experiments in Series I shows that changes concomitant with induction were no larger than spontaneous changes. **D **Comparison of spontaneous changes and changes concomitant with induction in hourly burst rates. Unlike in *C*, all changes were normalized to the hourly burst rate before the induction sequence. Data are mean ± SEM of absolute values of changes; *N *= 4, 16, 8 for protocols **I.1**, **I.2**, **I.3 **respectively. Paired t-tests revealed no significant effects of the induction sequence.

Only one experiment out of 28 showed significantly larger changes concomitant with the induction sequence than in spontaneous activity; this is the example shown in Figure 3A. Contrary to the observations by [24], these changes consisted of a decrease in burst rates. More typically, the induction sequences had no appreciable effect (see, for example, Figure 3B). Overall, changes concomitant with induction were no larger than spontaneous changes in any protocol (Figure 3C). It is not clear why one culture did show plasticity; apart from its reaction to the induction sequence, nothing set it obviously apart from its sister cultures. Certainly, the top panel of Figure 3A looks quite convincing, so it is attractive to hypothesize that something special happened. However, the culture used in this experiment was not in any way special: its age was in the middle of the range, we noted no distinguishing physical characteristics, and its pre-experimental activity was similar to the other cultures tested. Thus, we suspect the results may have been a statistical fluke. After all, testing at the *p *< .05-level, one positive result out of 28 is not unexpected.

For the purpose of comparing results between cultures with widely varying burst rates, we normalized the changes by the baseline burst rates *N*_base_, and calculated the averages of |Δ*N*_ind_|/*N*_base _and |Δ*N*_spont_|/*N*_base _across all experiments with a given protocol. This revealed that changes concomitant with the induction sequence were not significantly greater than spontaneous changes in any protocol (Figure 3D). (In protocol **I.3**, with elevated extracellular magnesium to reduce bursting, spontaneous changes were in fact larger. This may be due to transient effects of the magnesium, which partially wore off during the course of the experiment, resulting in additional drift, especially between control and baseline periods.) We also calculated the average number of spikes per burst before and after the induction sequence, and found no significant effects of stimulation in that measure either (data not shown).

### Series II: Changes induced in stimulus–response maps

We tested whether tetani delivered to individual electrodes could cause network-level plasticity resulting in changes in array-wide responses to probe stimuli on any electrode. As in Series I, several experiments were usually performed on each culture, with several hours between experiments.

#### Details of induction sequences

In most experiments, induction sequences consisted of several tetanic trains of stimuli delivered to a single electrode. Each train consisted of 20 pulses, at 50 ms intervals. A complete induction sequence consisted of 20 trains, with 2 s between trains. Before experiments, the relation between stimulation voltage and array-wide response strength was determined for each electrode (see *Choice of electrodes*, under *Methods*). For tetanization, we then chose electrodes that evoked strong culture-wide responses. In one set of experiments (protocols **II.5a **and **b**), tetanic stimulation was applied to clusters of electrodes, as in **I.3a **and **b**. Details of all experiments are summarized in Table 3.

**Table 3:** Details of experiments on plasticity expressed in stimulus–response maps (Series II).

**Protocol**	**Tetanus target**	**Probe amplitude**	**Conditions**	**No. and ages of cultures **	**Total expts.**	**Intervals**
**II.1**	Single electrode.	Fixed, 0.8 V.	Baseline medium, spontaneous bursting.	4; 17–22 div	8	2 h
**II.2**	Single electrode.	Fixed, 0.8 V.	Bursts completely suppressed by 50 Hz background stimulation distributed over 20–40 electrodes, except during tetanization.	3^*a*^; 17–22 div	6	2 h
**II.3**	Single electrode.	Fixed, 0.8 V.	Spontaneous bursts suppressed by 1 mM magnesium.	3; 26–28 div	6	2 h
**II.4**	Single electrode.	Reduced (see Methods).	Spontaneous bursts suppressed by 2 mM magnesium.	4; 29–32 div	16	2 h
**II.5a**	8 electrodes, as in **I.3a**.	Range of voltages, 100–900 mV.	Spontaneous bursts suppressed by 1–2 mM magnesium.	3; 18–20 div	12	2 h
**II.5b**	8 electrodes, as in **I.3b**.	Range of voltages, 100–900 mV.	Spontaneous burst suppressed by 2 mM magnesium.	1; 17 div	4	2 h

^*a *^In a 4th experiment, burst suppression did not work sufficiently well. Those data were excluded from further analysis.

#### Details of probe sequences

Each of the 59 electrodes in the array was probed with test stimuli for a one-hour "control" period followed by a one-hour "baseline" period. Probes were delivered cyclically to all electrodes, with 3 s between pulses. The firing rates of each of 58 functional recording electrodes were observed, 10–50 ms after a test pulse to one of the 59 stimulation electrodes. After the tetanic induction sequence, the network was probed in the same manner for another one-hour "posttetanic" period. In most experiments, probe pulse amplitudes were fixed at 0.8 V. In some (protocol **II.4), **they were reduced in an attempt to define probe pulses that would not evoke culture-wide bursts. (This attempt was largely unsuccessful, see *Methods*.) In protocols **II.5a **and **b **we probed for test responses using many different pulse amplitudes.

#### Data analysis and results

By averaging the responses recorded within each one-hour period (separately for each stimulation electrode-recording electrode pair), a response map was constructed. Differences between the "baseline" and "posttetanic" maps were then compared to differences between the "baseline" and "control" maps. Specifically, we counted the number of spikes 10–50 ms after each probe stimulus, separately for each recording electrode. For each of the three periods, we then computed the mean number of spikes detected on electrode *R *(for 'Recording'), after a test stimulus on electrode *S *(for 'Stimulation'): nbaseSR
 MathType@MTEF@5@5@+=feaafiart1ev1aaatCvAUfKttLearuWrP9MDH5MBPbIqV92AaeXatLxBI9gBamXvP5wqSXMqHnxAJn0BKvguHDwzZbqegyvzYrwyUfgarqqtubsr4rNCHbGeaGqiA8vkIkVAFgIELiFeLkFeLk=iY=Hhbbf9v8qqaqFr0xc9pk0xbba9q8WqFfeaY=biLkVcLq=JHqVepeea0=as0db9vqpepesP0xe9Fve9Fve9GapdbaqaaeGacaGaaiaabeqaamqadiabaaGcbaGaemOBa42aa0baaSqaaiabbkgaIjabbggaHjabbohaZjabbwgaLbqaaiabdofatjabdkfasbaaaaa@462B@ (the mean over all stimuli to a given electrode *S* in the baseline period just before tetanization), nctrlSR
 MathType@MTEF@5@5@+=feaafiart1ev1aaatCvAUfKttLearuWrP9MDH5MBPbIqV92AaeXatLxBI9gBamXvP5wqSXMqHnxAJn0BKvguHDwzZbqegyvzYrwyUfgarqqtubsr4rNCHbGeaGqiA8vkIkVAFgIELiFeLkFeLk=iY=Hhbbf9v8qqaqFr0xc9pk0xbba9q8WqFfeaY=biLkVcLq=JHqVepeea0=as0db9vqpepesP0xe9Fve9Fve9GapdbaqaaeGacaGaaiaabeqaamqadiabaaGcbaGaemOBa42aa0baaSqaaiabbogaJjabbsha0jabbkhaYjabbYgaSbqaaiabdofatjabdkfasbaaaaa@465F@ (the means for the control period before that), and npostSR
 MathType@MTEF@5@5@+=feaafiart1ev1aaatCvAUfKttLearuWrP9MDH5MBPbIqV92AaeXatLxBI9gBamXvP5wqSXMqHnxAJn0BKvguHDwzZbqegyvzYrwyUfgarqqtubsr4rNCHbGeaGqiA8vkIkVAFgIELiFeLkFeLk=iY=Hhbbf9v8qqaqFr0xc9pk0xbba9q8WqFfeaY=biLkVcLq=JHqVepeea0=as0db9vqpepesP0xe9Fve9Fve9GapdbaqaaeGacaGaaiaabeqaamqadiabaaGcbaGaemOBa42aa0baaSqaaiabbchaWjabb+gaVjabbohaZjabbsha0bqaaiabdofatjabdkfasbaaaaa@4681@ (the means for the hour immediately after tetanization).

We wanted to know not only whether significant tetanus-related changes occurred in individual (S,*R*)-pairs, but also whether such changes were linked to specific stimulation sites, as reported in [26]. In that case, responses on all or most recording sites to one given stimulation site should be up- or downregulated together. We also considered the converse hypothesis: changes might occur at specific recording sites, in other words, all responses on a given recording site could be up- or downregulated together, independently of which stimulation site was used to evoke the response. To test these hypotheses, we calculated

Δnindstim≡∑S|∑R(npostSR−nbaseSR)|,
 MathType@MTEF@5@5@+=feaafiart1ev1aaatCvAUfKttLearuWrP9MDH5MBPbIqV92AaeXatLxBI9gBamXvP5wqSXMqHnxAJn0BKvguHDwzZbqegyvzYrwyUfgarqqtubsr4rNCHbGeaGqiA8vkIkVAFgIELiFeLkFeLk=iY=Hhbbf9v8qqaqFr0xc9pk0xbba9q8WqFfeaY=biLkVcLq=JHqVepeea0=as0db9vqpepesP0xe9Fve9Fve9GapdbaqaaeGacaGaaiaabeqaamqadiabaaGcbaGaeuiLdqKaemOBa42aa0baaSqaaiabbMgaPjabb6gaUjabbsgaKbqaaiabbohaZjabbsha0jabbMgaPjabb2gaTbaakiabggMi6oaaqafabaWaaqWaaeaadaaeqbqaamaabmaabaGaemOBa42aa0baaSqaaiabbchaWjabb+gaVjabbohaZjabbsha0bqaaiabdofatjabdkfasbaakiabgkHiTiabd6gaUnaaDaaaleaacqqGIbGycqqGHbqycqqGZbWCcqqGLbqzaeaacqWGtbWucqWGsbGuaaaakiaawIcacaGLPaaaaSqaaiabdkfasbqab0GaeyyeIuoaaOGaay5bSlaawIa7aaWcbaGaem4uamfabeqdcqGHris5aOGaeiilaWcaaa@6B6F@

which would deviate significantly from zero if changes were stimulation-site specific (as in [26]), as well as

Δnindrec≡∑R|∑S(npostSR−nbaseSR)|,
 MathType@MTEF@5@5@+=feaafiart1ev1aaatCvAUfKttLearuWrP9MDH5MBPbIqV92AaeXatLxBI9gBamXvP5wqSXMqHnxAJn0BKvguHDwzZbqegyvzYrwyUfgarqqtubsr4rNCHbGeaGqiA8vkIkVAFgIELiFeLkFeLk=iY=Hhbbf9v8qqaqFr0xc9pk0xbba9q8WqFfeaY=biLkVcLq=JHqVepeea0=as0db9vqpepesP0xe9Fve9Fve9GapdbaqaaeGacaGaaiaabeqaamqadiabaaGcbaGaeuiLdqKaemOBa42aa0baaSqaaiabbMgaPjabb6gaUjabbsgaKbqaaiabbkhaYjabbwgaLjabbogaJbaakiabggMi6oaaqafabaWaaqWaaeaadaaeqbqaamaabmaabaGaemOBa42aa0baaSqaaiabbchaWjabb+gaVjabbohaZjabbsha0bqaaiabdofatjabdkfasbaakiabgkHiTiabd6gaUnaaDaaaleaacqqGIbGycqqGHbqycqqGZbWCcqqGLbqzaeaacqWGtbWucqWGsbGuaaaakiaawIcacaGLPaaaaSqaaiabdofatbqab0GaeyyeIuoaaOGaay5bSlaawIa7aaWcbaGaemOuaifabeqdcqGHris5aOGaeiilaWcaaa@69E2@

which would deviate significantly from zero if changes were recording-site specific. (If changes were randomly distributed, both inner sums would have a roughly equal number of positive and negative terms, and hence not be very large.)

**Figure 4:**
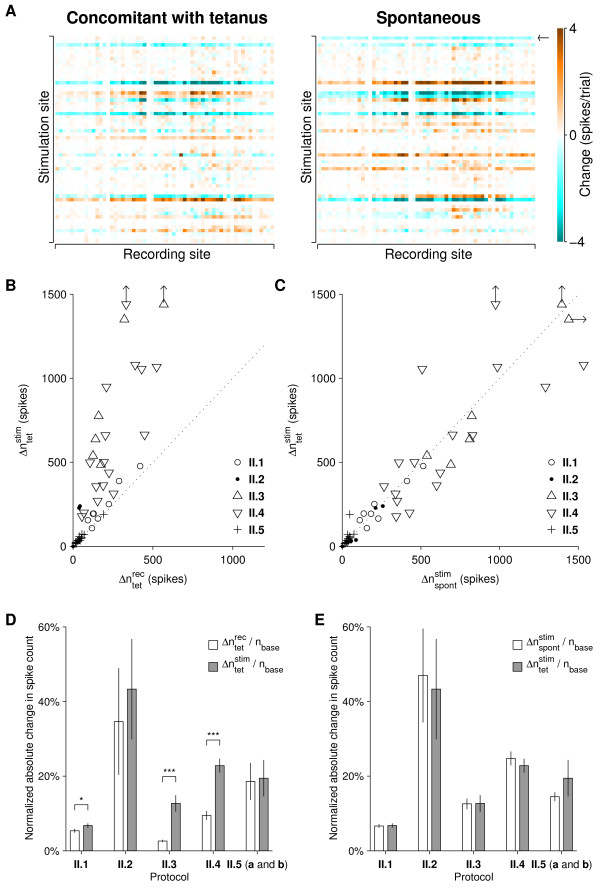
**Results of Series II: Experiments on plasticity expressed in stimulus–response maps**. **A **An example from protocol **II.3**. Colored pixels represent changes in the average number of spikes on a given recording electrode 10–50 ms after a test pulse to a given stimulation electrode. The horizontal stripes of similar coloration reveal stimulation-site-specific changes. However, spontaneous changes (*right*) were comparable in magnitude to changes concomitant with tetani (*left*). **B **A direct comparison between stimulation-site-specific changes and recording-site-specific changes across tetani reveals that stimulation-site-specific changes were dominant in all experiments. Each point corresponds to one experiment. Plot symbols indicate tetanization protocols; arrows mark data points that fell outside the plot limits. **C **Direct comparison between stimulation-site-specific changes concomitant with tetanization and due to spontaneous drift reveals that tetanization does not cause enhanced change compared to drift. **D **Summary of data in *B*. All values were normalized by nbase≡∑S,RnbaseSR
 MathType@MTEF@5@5@+=feaafiart1ev1aaatCvAUfKttLearuWrP9MDH5MBPbIqV92AaeXatLxBI9gBamXvP5wqSXMqHnxAJn0BKvguHDwzZbqegyvzYrwyUfgarqqtubsr4rNCHbGeaGqiA8vkIkVAFgIELiFeLkFeLk=iY=Hhbbf9v8qqaqFr0xc9pk0xbba9q8WqFfeaY=biLkVcLq=JHqVepeea0=as0db9vqpepesP0xe9Fve9Fve9GapdbaqaaeGacaGaaiaabeqaamqadiabaaGcbaGaemOBa42aaSbaaSqaaiabbkgaIjabbggaHjabbohaZjabbwgaLbqabaGccqGHHjIUdaaeqaqaaiabd6gaUnaaDaaaleaacqqGIbGycqqGHbqycqqGZbWCcqqGLbqzaeaacqWGtbWucqWGsbGuaaaabaGaem4uamLaeiilaWIaemOuaifabeqdcqGHris5aaaa@53F5@. Asterisks indicate significance: *p *< 0.05 (*) or *p *< 0.001 (***), two-tailed t-test, *N *= 8, 6, 6, 16, 16 for protocols **II.1, II.2, II.5. E **Summary of data in *C*, same normalization as in *D*. T-tests revealed no significant effects of tetanization.

In protocols **II.3 **and **II.4**, stimulation-site-specific changes exceeded recording-site-specific changes, in agreement with [26]; see Figure 4A for an example. However, stimulation-site-specific differences between the control and baseline periods were also observed, and no obvious difference was seen between the spontaneous differences and those concomitant with tetanization. We quantified this by calculating

Δnspontstim≡∑S|∑R(nbaseSR−nctrlSR)|,
 MathType@MTEF@5@5@+=feaafiart1ev1aaatCvAUfKttLearuWrP9MDH5MBPbIqV92AaeXatLxBI9gBamXvP5wqSXMqHnxAJn0BKvguHDwzZbqegyvzYrwyUfgarqqtubsr4rNCHbGeaGqiA8vkIkVAFgIELiFeLkFeLk=iY=Hhbbf9v8qqaqFr0xc9pk0xbba9q8WqFfeaY=biLkVcLq=JHqVepeea0=as0db9vqpepesP0xe9Fve9Fve9GapdbaqaaeGacaGaaiaabeqaamqadiabaaGcbaGaeuiLdqKaemOBa42aa0baaSqaaiabbohaZjabbchaWjabb+gaVjabb6gaUjabbsha0bqaaiabbohaZjabbsha0jabbMgaPjabb2gaTbaakiabggMi6oaaqafabaWaaqWaaeaadaaeqbqaamaabmaabaGaemOBa42aa0baaSqaaiabbkgaIjabbggaHjabbohaZjabbwgaLbqaaiabdofatjabdkfasbaakiabgkHiTiabd6gaUnaaDaaaleaacqqGJbWycqqG0baDcqqGYbGCcqqGSbaBaeaacqWGtbWucqWGsbGuaaaakiaawIcacaGLPaaaaSqaaiabdkfasbqab0GaeyyeIuoaaOGaay5bSlaawIa7aaWcbaGaem4uamfabeqdcqGHris5aOGaeiilaWcaaa@6E4D@

and comparing this with Δnindstim
 MathType@MTEF@5@5@+=feaafiart1ev1aaatCvAUfKttLearuWrP9MDH5MBPbIqV92AaeXatLxBI9gBamXvP5wqSXMqHnxAJn0BKvguHDwzZbqegyvzYrwyUfgarqqtubsr4rNCHbGeaGqiA8vkIkVAFgIELiFeLkFeLk=iY=Hhbbf9v8qqaqFr0xc9pk0xbba9q8WqFfeaY=biLkVcLq=JHqVepeea0=as0db9vqpepesP0xe9Fve9Fve9GapdbaqaaeGacaGaaiaabeqaamqadiabaaGcbaGaeuiLdqKaemOBa42aa0baaSqaaiabbMgaPjabb6gaUjabbsgaKbqaaiabbohaZjabbsha0jabbMgaPjabb2gaTbaaaaa@4984@. In protocols **II.5a **and **b**, where stimuli of many different voltages were used on each electrode, we considered each of the ~3400 stimulus–response pairs in turn, and fitted a straight line to the response 10–50 ms post-stimulus vs. voltage, independently for each hour. The fit value at 700 mV was then compared before and after the induction sequence, just as *n*^*SR *^was in other protocols. While Δnindstim
 MathType@MTEF@5@5@+=feaafiart1ev1aaatCvAUfKttLearuWrP9MDH5MBPbIqV92AaeXatLxBI9gBamXvP5wqSXMqHnxAJn0BKvguHDwzZbqegyvzYrwyUfgarqqtubsr4rNCHbGeaGqiA8vkIkVAFgIELiFeLkFeLk=iY=Hhbbf9v8qqaqFr0xc9pk0xbba9q8WqFfeaY=biLkVcLq=JHqVepeea0=as0db9vqpepesP0xe9Fve9Fve9GapdbaqaaeGacaGaaiaabeqaamqadiabaaGcbaGaeuiLdqKaemOBa42aa0baaSqaaiabbMgaPjabb6gaUjabbsgaKbqaaiabbohaZjabbsha0jabbMgaPjabb2gaTbaaaaa@4984@ differed significantly from Δnindrec
 MathType@MTEF@5@5@+=feaafiart1ev1aaatCvAUfKttLearuWrP9MDH5MBPbIqV92AaeXatLxBI9gBamXvP5wqSXMqHnxAJn0BKvguHDwzZbqegyvzYrwyUfgarqqtubsr4rNCHbGeaGqiA8vkIkVAFgIELiFeLkFeLk=iY=Hhbbf9v8qqaqFr0xc9pk0xbba9q8WqFfeaY=biLkVcLq=JHqVepeea0=as0db9vqpepesP0xe9Fve9Fve9GapdbaqaaeGacaGaaiaabeqaamqadiabaaGcbaGaeuiLdqKaemOBa42aa0baaSqaaiabbMgaPjabb6gaUjabbsgaKbqaaiabbkhaYjabbwgaLjabbogaJbaaaaa@47F7@ in protocols **II.3 **and **II.4 **(Figure 4B and 4D), it did not differ significantly from Δnspontstim
 MathType@MTEF@5@5@+=feaafiart1ev1aaatCvAUfKttLearuWrP9MDH5MBPbIqV92AaeXatLxBI9gBamXvP5wqSXMqHnxAJn0BKvguHDwzZbqegyvzYrwyUfgarqqtubsr4rNCHbGeaGqiA8vkIkVAFgIELiFeLkFeLk=iY=Hhbbf9v8qqaqFr0xc9pk0xbba9q8WqFfeaY=biLkVcLq=JHqVepeea0=as0db9vqpepesP0xe9Fve9Fve9GapdbaqaaeGacaGaaiaabeqaamqadiabaaGcbaGaeuiLdqKaemOBa42aa0baaSqaaiabbohaZjabbchaWjabb+gaVjabb6gaUjabbsha0bqaaiabbohaZjabbsha0jabbMgaPjabb2gaTbaaaaa@4C84@ (Figure 4C and 4E). Thus, the stimulation-site-specific changes could not be attributed to the tetanization. In protocol **II.1 **stimulation-site-specific changes across tetani were also slightly larger than recording-site-specific changes, *but *again they were no larger than spontaneous changes. In protocols **II.2 **and **II.5 **no significant effects were seen at all. In short, no interesting changes could be attributed to the induction sequences in any of the experiments in Series II. (As an aside, extending the response window to 10–160 ms (as in [26]) did not improve statistics; we found that probe responses were typically largely over before 50 ms poststimulus, so lengthening the window mainly added background activity to the spike counts.)

#### Changes in the probability of evoking bursts

In addition to evoking immediate responses, electrical stimulation can often evoke bursts [33]. Therefore, in addition to testing for changes induced in stimulus-response maps, we investigated whether tetanization had an effect on the ability of test pulses to evoke bursts. We counted spikes across the array 100–500 ms after each stimulus, and found a clearly bimodal distribution in each experiment, making it very easy to distinguish trials that evoked bursts from those that did not. For each stimulation electrode, we determined the fraction of stimuli that evoked bursts in one-hour windows. We calculated spontaneous changes and changes concomitant with tetani in this fraction, and found that they were equally large (data not shown). In conclusion, tetanization did not affect the probability of evoking bursts by test pulses.

#### The origin of apparent stimulation-site specific changes

**Figure 5:**
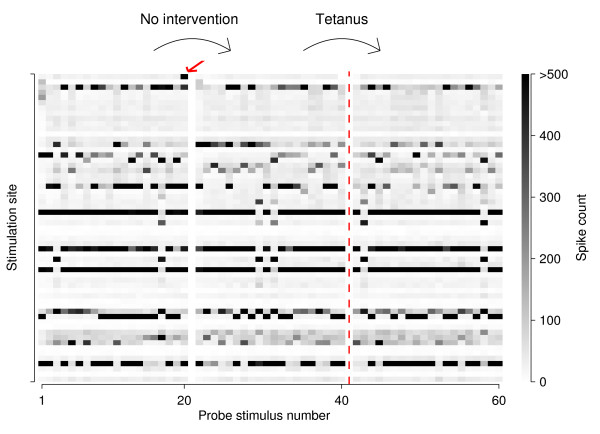
**Array-wide responses to individual stimuli in an experiment from protocol II.3**. Spikes recorded on any electrode, 10–50 ms after a stimulus, are counted together and represented on a gray scale. Each pixel is one stimulus response; stimulation order is top to bottom, then left to right. Separating white bars indicate division into 1-hr windows (corresponding to blocks of 20 trials each per stimulation electrode); the dashed line represents the tetanus. Stimuli that evoked bursts show up as black pixels. The red arrow points to a burst that, on its own, was responsible for the top-most horizontal blue stripe in Figure 4A, right sub-panel.

How can apparently highly significant stimulation-site-specific changes result spontaneously? We hypothesized that network-wide bursts played an important role, since they occurred only in some fraction of trials, but could contribute strongly to the total number of spikes recorded in those trials, and hence could be a major source of variability. Consider, for instance, a typical experiment from protocol **II.3. **In the window of 10–50 ms post-stimulus used for quantifying responses, most probe pulses elicited a total of around 10–100 spikes across the entire recording array, if no burst was evoked. If a burst *was *evoked, this number could easily exceed 500 or even 1000, so bursts have a major impact on the average spike count across trials. Array-wide spike counts recorded after multiple individual stimuli to each of 59 electrodes are shown in Figure 5 for the same experiment that served as an example in Figure 4A. It shows an immediately obvious dichotomy between stimuli that elicited bursts and those that did not: the former are represented by black or very dark pixels, the latter by light gray pixels. While stimuli to certain electrodes more readily evoked bursts than others, burst generation was never quite reliable, so if (for example) 4 out of 20 stimuli to a given electrode evoked bursts in one hour, that number might well be 3 or 5 in the next hour. Since network-wide bursts by definition affect all or most recording sites, a random increase (or decrease) from one hour to the next in the number of bursts evoked by stimuli to an electrode *S *will result in changes in *n*^*SR *^that have the same sign for all recording electrodes *R*. This produces a uniformly brown (or blue) stripe in the representation of Figure 4A, and contributes a large positive term to Δnindstim
 MathType@MTEF@5@5@+=feaafiart1ev1aaatCvAUfKttLearuWrP9MDH5MBPbIqV92AaeXatLxBI9gBamXvP5wqSXMqHnxAJn0BKvguHDwzZbqegyvzYrwyUfgarqqtubsr4rNCHbGeaGqiA8vkIkVAFgIELiFeLkFeLk=iY=Hhbbf9v8qqaqFr0xc9pk0xbba9q8WqFfeaY=biLkVcLq=JHqVepeea0=as0db9vqpepesP0xe9Fve9Fve9GapdbaqaaeGacaGaaiaabeqaamqadiabaaGcbaGaeuiLdqKaemOBa42aa0baaSqaaiabbMgaPjabb6gaUjabbsgaKbqaaiabbohaZjabbsha0jabbMgaPjabb2gaTbaaaaa@4984@ or Δnspontstim
 MathType@MTEF@5@5@+=feaafiart1ev1aaatCvAUfKttLearuWrP9MDH5MBPbIqV92AaeXatLxBI9gBamXvP5wqSXMqHnxAJn0BKvguHDwzZbqegyvzYrwyUfgarqqtubsr4rNCHbGeaGqiA8vkIkVAFgIELiFeLkFeLk=iY=Hhbbf9v8qqaqFr0xc9pk0xbba9q8WqFfeaY=biLkVcLq=JHqVepeea0=as0db9vqpepesP0xe9Fve9Fve9GapdbaqaaeGacaGaaiaabeqaamqadiabaaGcbaGaeuiLdqKaemOBa42aa0baaSqaaiabbohaZjabbchaWjabb+gaVjabb6gaUjabbsha0bqaaiabbohaZjabbsha0jabbMgaPjabb2gaTbaaaaa@4C84@ (Whether the effect is on Δnindstim
 MathType@MTEF@5@5@+=feaafiart1ev1aaatCvAUfKttLearuWrP9MDH5MBPbIqV92AaeXatLxBI9gBamXvP5wqSXMqHnxAJn0BKvguHDwzZbqegyvzYrwyUfgarqqtubsr4rNCHbGeaGqiA8vkIkVAFgIELiFeLkFeLk=iY=Hhbbf9v8qqaqFr0xc9pk0xbba9q8WqFfeaY=biLkVcLq=JHqVepeea0=as0db9vqpepesP0xe9Fve9Fve9GapdbaqaaeGacaGaaiaabeqaamqadiabaaGcbaGaeuiLdqKaemOBa42aa0baaSqaaiabbMgaPjabb6gaUjabbsgaKbqaaiabbohaZjabbsha0jabbMgaPjabb2gaTbaaaaa@4984@ or on Δnspontstim
 MathType@MTEF@5@5@+=feaafiart1ev1aaatCvAUfKttLearuWrP9MDH5MBPbIqV92AaeXatLxBI9gBamXvP5wqSXMqHnxAJn0BKvguHDwzZbqegyvzYrwyUfgarqqtubsr4rNCHbGeaGqiA8vkIkVAFgIELiFeLkFeLk=iY=Hhbbf9v8qqaqFr0xc9pk0xbba9q8WqFfeaY=biLkVcLq=JHqVepeea0=as0db9vqpepesP0xe9Fve9Fve9GapdbaqaaeGacaGaaiaabeqaamqadiabaaGcbaGaeuiLdqKaemOBa42aa0baaSqaaiabbohaZjabbchaWjabb+gaVjabb6gaUjabbsha0bqaaiabbohaZjabbsha0jabbMgaPjabb2gaTbaaaaa@4C84@ depends on when the random increase or decrease occurred. Either can happen with equal likelihood, because the effect is unrelated to tetanization). To give a specific example, the burst response indicated by the small arrow in Figure 5 is responsible for the blue row of pixels across the top of the right panel of Figure 4A. By contrast, the response to 59 × 20 = 1180 stimuli are combined in the inner sum of Δnindrec
 MathType@MTEF@5@5@+=feaafiart1ev1aaatCvAUfKttLearuWrP9MDH5MBPbIqV92AaeXatLxBI9gBamXvP5wqSXMqHnxAJn0BKvguHDwzZbqegyvzYrwyUfgarqqtubsr4rNCHbGeaGqiA8vkIkVAFgIELiFeLkFeLk=iY=Hhbbf9v8qqaqFr0xc9pk0xbba9q8WqFfeaY=biLkVcLq=JHqVepeea0=as0db9vqpepesP0xe9Fve9Fve9GapdbaqaaeGacaGaaiaabeqaamqadiabaaGcbaGaeuiLdqKaemOBa42aa0baaSqaaiabbMgaPjabb6gaUjabbsgaKbqaaiabbkhaYjabbwgaLjabbogaJbaaaaa@47F7@ (as well as Δnspontrec
 MathType@MTEF@5@5@+=feaafiart1ev1aaatCvAUfKttLearuWrP9MDH5MBPbIqV92AaeXatLxBI9gBamXvP5wqSXMqHnxAJn0BKvguHDwzZbqegyvzYrwyUfgarqqtubsr4rNCHbGeaGqiA8vkIkVAFgIELiFeLkFeLk=iY=Hhbbf9v8qqaqFr0xc9pk0xbba9q8WqFfeaY=biLkVcLq=JHqVepeea0=as0db9vqpepesP0xe9Fve9Fve9GapdbaqaaeGacaGaaiaabeqaamqadiabaaGcbaGaeuiLdqKaemOBa42aa0baaSqaaiabbohaZjabbchaWjabb+gaVjabb6gaUjabbsha0bqaaiabbkhaYjabbwgaLjabbogaJbaaaaa@4AF7@), so individual bursts make a much smaller relative contribution to those measures. Moreover, when one electrode elicits an extra burst in a given hour, another electrode is likely to elicit one burst fewer, further reducing their net contribution to Δnindrec
 MathType@MTEF@5@5@+=feaafiart1ev1aaatCvAUfKttLearuWrP9MDH5MBPbIqV92AaeXatLxBI9gBamXvP5wqSXMqHnxAJn0BKvguHDwzZbqegyvzYrwyUfgarqqtubsr4rNCHbGeaGqiA8vkIkVAFgIELiFeLkFeLk=iY=Hhbbf9v8qqaqFr0xc9pk0xbba9q8WqFfeaY=biLkVcLq=JHqVepeea0=as0db9vqpepesP0xe9Fve9Fve9GapdbaqaaeGacaGaaiaabeqaamqadiabaaGcbaGaeuiLdqKaemOBa42aa0baaSqaaiabbMgaPjabb6gaUjabbsgaKbqaaiabbkhaYjabbwgaLjabbogaJbaaaaa@47F7@ and Δnspontrec
 MathType@MTEF@5@5@+=feaafiart1ev1aaatCvAUfKttLearuWrP9MDH5MBPbIqV92AaeXatLxBI9gBamXvP5wqSXMqHnxAJn0BKvguHDwzZbqegyvzYrwyUfgarqqtubsr4rNCHbGeaGqiA8vkIkVAFgIELiFeLkFeLk=iY=Hhbbf9v8qqaqFr0xc9pk0xbba9q8WqFfeaY=biLkVcLq=JHqVepeea0=as0db9vqpepesP0xe9Fve9Fve9GapdbaqaaeGacaGaaiaabeqaamqadiabaaGcbaGaeuiLdqKaemOBa42aa0baaSqaaiabbohaZjabbchaWjabb+gaVjabb6gaUjabbsha0bqaaiabbkhaYjabbwgaLjabbogaJbaaaaa@4AF7@.

The effect of network-wide bursts was greatest in protocol **II.3–5**, probably because elevated magnesium caused bursts to be relatively rare and discrete events. In baseline medium (protocol **II.1), **bursts occurred much more frequently, while individual bursts were smaller and more variable in size, reducing their impact on Δnindstim
 MathType@MTEF@5@5@+=feaafiart1ev1aaatCvAUfKttLearuWrP9MDH5MBPbIqV92AaeXatLxBI9gBamXvP5wqSXMqHnxAJn0BKvguHDwzZbqegyvzYrwyUfgarqqtubsr4rNCHbGeaGqiA8vkIkVAFgIELiFeLkFeLk=iY=Hhbbf9v8qqaqFr0xc9pk0xbba9q8WqFfeaY=biLkVcLq=JHqVepeea0=as0db9vqpepesP0xe9Fve9Fve9GapdbaqaaeGacaGaaiaabeqaamqadiabaaGcbaGaeuiLdqKaemOBa42aa0baaSqaaiabbMgaPjabb6gaUjabbsgaKbqaaiabbohaZjabbsha0jabbMgaPjabb2gaTbaaaaa@4984@ and Δnspontstim
 MathType@MTEF@5@5@+=feaafiart1ev1aaatCvAUfKttLearuWrP9MDH5MBPbIqV92AaeXatLxBI9gBamXvP5wqSXMqHnxAJn0BKvguHDwzZbqegyvzYrwyUfgarqqtubsr4rNCHbGeaGqiA8vkIkVAFgIELiFeLkFeLk=iY=Hhbbf9v8qqaqFr0xc9pk0xbba9q8WqFfeaY=biLkVcLq=JHqVepeea0=as0db9vqpepesP0xe9Fve9Fve9GapdbaqaaeGacaGaaiaabeqaamqadiabaaGcbaGaeuiLdqKaemOBa42aa0baaSqaaiabbohaZjabbchaWjabb+gaVjabb6gaUjabbsha0bqaaiabbohaZjabbsha0jabbMgaPjabb2gaTbaaaaa@4C84@ . With electrical burst quieting, bursts did not usually occur at all, and could therefore not contribute to apparent plasticity. Indeed, in protocol **II.2**, Δnindstim
 MathType@MTEF@5@5@+=feaafiart1ev1aaatCvAUfKttLearuWrP9MDH5MBPbIqV92AaeXatLxBI9gBamXvP5wqSXMqHnxAJn0BKvguHDwzZbqegyvzYrwyUfgarqqtubsr4rNCHbGeaGqiA8vkIkVAFgIELiFeLkFeLk=iY=Hhbbf9v8qqaqFr0xc9pk0xbba9q8WqFfeaY=biLkVcLq=JHqVepeea0=as0db9vqpepesP0xe9Fve9Fve9GapdbaqaaeGacaGaaiaabeqaamqadiabaaGcbaGaeuiLdqKaemOBa42aa0baaSqaaiabbMgaPjabb6gaUjabbsgaKbqaaiabbohaZjabbsha0jabbMgaPjabb2gaTbaaaaa@4984@ and Δnspontstim
 MathType@MTEF@5@5@+=feaafiart1ev1aaatCvAUfKttLearuWrP9MDH5MBPbIqV92AaeXatLxBI9gBamXvP5wqSXMqHnxAJn0BKvguHDwzZbqegyvzYrwyUfgarqqtubsr4rNCHbGeaGqiA8vkIkVAFgIELiFeLkFeLk=iY=Hhbbf9v8qqaqFr0xc9pk0xbba9q8WqFfeaY=biLkVcLq=JHqVepeea0=as0db9vqpepesP0xe9Fve9Fve9GapdbaqaaeGacaGaaiaabeqaamqadiabaaGcbaGaeuiLdqKaemOBa42aa0baaSqaaiabbohaZjabbchaWjabb+gaVjabb6gaUjabbsha0bqaaiabbohaZjabbsha0jabbMgaPjabb2gaTbaaaaa@4C84@ were not significantly different from Δnindrec
 MathType@MTEF@5@5@+=feaafiart1ev1aaatCvAUfKttLearuWrP9MDH5MBPbIqV92AaeXatLxBI9gBamXvP5wqSXMqHnxAJn0BKvguHDwzZbqegyvzYrwyUfgarqqtubsr4rNCHbGeaGqiA8vkIkVAFgIELiFeLkFeLk=iY=Hhbbf9v8qqaqFr0xc9pk0xbba9q8WqFfeaY=biLkVcLq=JHqVepeea0=as0db9vqpepesP0xe9Fve9Fve9GapdbaqaaeGacaGaaiaabeqaamqadiabaaGcbaGaeuiLdqKaemOBa42aa0baaSqaaiabbMgaPjabb6gaUjabbsgaKbqaaiabbkhaYjabbwgaLjabbogaJbaaaaa@47F7@ and Δnspontrec
 MathType@MTEF@5@5@+=feaafiart1ev1aaatCvAUfKttLearuWrP9MDH5MBPbIqV92AaeXatLxBI9gBamXvP5wqSXMqHnxAJn0BKvguHDwzZbqegyvzYrwyUfgarqqtubsr4rNCHbGeaGqiA8vkIkVAFgIELiFeLkFeLk=iY=Hhbbf9v8qqaqFr0xc9pk0xbba9q8WqFfeaY=biLkVcLq=JHqVepeea0=as0db9vqpepesP0xe9Fve9Fve9GapdbaqaaeGacaGaaiaabeqaamqadiabaaGcbaGaeuiLdqKaemOBa42aa0baaSqaaiabbohaZjabbchaWjabb+gaVjabb6gaUjabbsha0bqaaiabbkhaYjabbwgaLjabbogaJbaaaaa@4AF7@. (Note that the relative changes shown in Figure 4D and 4E were larger for protocol **II.2 **than for the other protocols overall. This was due to a smaller absolute spike count in probe responses in the presence of quieting stimuli.)

### Series III: Changes in specific responses

Paired-pulse stimulation of a presynaptic and a postsynaptic cell with sharp intracellular or patch electrodes is a well established protocol for inducing plasticity. Depending on the timing between the pulses, both long-term potentiation (LTP) and long-term depression (LTD) can be obtained using this technique in cultures from many brain regions, including cortex and hippocampus [35,17]. We tested whether a similar protocol could be used with MEA electrodes. Since we have no direct information about which specific cells are stimulated by a given electrode, nor from which cells an electrode records, the search for modifiable synapses has to be, to some degree, blind. We reasoned that if test pulses to electrode *S*_1 _and test pulses to electrode *S*_2 _both evoked responses at electrode *R*, those responses would likely be affected if paired-pulse stimulation of *S*_1 _and *S*_2 _modified any synapses. This directed search for plasticity is more sensitive than a full (undirected) assay of changes in responses anywhere evoked by stimulation through any electrode, because for a full assay so many statistical tests have to be performed that a very tight probability bound must be used to avoid a deluge of false positives.

As before, experiments consisted of a one-hour long "control" period, followed by a one-hour long "baseline" period, followed by an induction sequence, and finally a one-hour long "post-induction" period. In each period, probe pulses were delivered to the tetanized electrodes *S*_1 _and *S*_2_, and to two control electrodes, sequentially and repeatedly. Responses (10–50 ms poststimulus) were recorded on 5–10 electrodes *R*_*i *_that, during pre-experimental probing, exhibited significantly elevated firing rates both in response to stimuli to *S*_1 _and in response to stimuli to *S*_2_.

#### Selecting stimulation pairs

To find pairs of electrodes that shared synaptic targets, we started with the set of candidate stimulation electrodes identified in initial probing (see *Choice of electrodes*, under *Methods*), and delivered 50 pulses of fixed amplitude to each of them at 3 s intervals. For each stimulation electrode, we determined the set of recording electrodes that responded with a spike rate elevated above baseline by at least 5 times the standard deviation of the baseline, 10–50 ms post-stimulus. We then selected from about 2000 available stimulation pairs according to the following criteria:

Common response sites

Pairs of stimulation electrodes that evoked responses on many common target sites were preferred, because we hypothesized that plasticity would preferentially occur at those common targets.

Mutually exclusive response sites

Pairs of electrodes that *only *evoked responses on common target sites were not selected, to avoid the case where the pair of electrical pulses simply stimulated one common cell at different locations along its axon.

For each experiment, we adjusted the selection criteria until the number of candidate stimulation pairs was between 10 and 20. Each of these candidate pairs typically evoked responses at 5–20 shared electrodes, and there typically were 5–10 electrodes that responded to one but not the other member of the pair. For experimental use, we then selected three pairs such that the shared targets of any one pair minimally overlapped with the targets of the other pairs. The distance between stimulation electrodes within a pair was not a consideration, but no electrode could serve in more than one pair. Pairs one and two were tetanized in separate experiments, two hours apart; the third pair served as a control for both experiments.

### Details of induction sequences

Tetanization consisted of trains of pulse pairs: one pulse to each of two electrodes *(S*_1 _and *S*_2_), with 5 or 10 ms between pulses (inter-electrode interval; IEI). Each train consisted of 20 pairs, with 50 or 100 ms between pairs (inter-pair interval; IPI). A complete tetanization sequence contained 20 or 150 trains, at 2 or 6 s intervals (inter-train interval; ITI). During tetanization, quieting with electrical stimulation was suspended, except in protocol **III.4 **(Table 4 lists details of each of the four protocols in this series).

**Table 4:** Details of experiments on plasticity induced in specific responses (Series III)

**Protocol**	**Tetanus**	**Probing**	**Conditions**	**No. and ages of cultures**	**Total Expts.**	**Intervals**
**III.1**	20 trains (ITI: 2 s) of 20 pulse pairs (IPI: 50 ms; IEI: 5 ms).	Single pulses of 0.8 V, at 5 s intervals.	Baseline medium, with spontaneous bursts.	4; 13–16 div	8	2 h
**III.2**	20 trains (ITI: 2 s) of 20 pulse pairs (IPI: 50 ms; IEI: 5 ms).	Single pulses of 0.8 V, at 5 s intervals.	Electrical burst quieting as in **II.2**.	3^*a*^; 13–16 div	6	2 h
**III.3**	20 trains (ITI: 2 s) of 20 pulse pairs (IPI: 50 ms; IEI: 5 ms).	Single pulses of 0.8 V, at 5 s intervals.	Spontaneous bursts suppressed by 1 mM magnesium.	4; 25–28 div	8	2 h
**III.4**	150 trains (ITI: 6 s) of 20 pulse pairs (IPI: 100 ms; IEI: 10 ms).	Single pulses of 0.6 V, at 1 s intervals.	Electrical burst quieting as in **II.2**, but not suspended during tetanization.	4; 20–23 div	8	2 h

^*a *^In a 4th culture, burst suppression did not work sufficiently well. Those data were excluded from further analysis.

#### Details of probe sequences

Test pulses with fixed amplitude (0.6 or 0.8 V) were delivered to each of the selected stimulation electrodes. Test pulses were presented in cyclic order, with 1 or 5 s between pulses (so that each electrode was stimulated once every 6 or 30 s). Where electrical burst quieting was used, quieting was suspended for 50 ms before and 200 ms after a test pulse, so that the responses to test pulses could be measured without interference. While all six electrodes were probed during both experiments, only responses to the two tetanized electrodes and the two controls were analyzed.

#### Data analysis and results

For each pair of stimulation electrode *S *and recording electrode *R *the average number of spikes *n*^*SR *^per probe response was determined in each period (control, baseline, and postinduction). The changes in those numbers concomitant with tetanization,

ΔnindSR≡npostSR−nbaseSR,
 MathType@MTEF@5@5@+=feaafiart1ev1aaatCvAUfKttLearuWrP9MDH5MBPbIqV92AaeXatLxBI9gBamXvP5wqSXMqHnxAJn0BKvguHDwzZbqegyvzYrwyUfgarqqtubsr4rNCHbGeaGqiA8vkIkVAFgIELiFeLkFeLk=iY=Hhbbf9v8qqaqFr0xc9pk0xbba9q8WqFfeaY=biLkVcLq=JHqVepeea0=as0db9vqpepesP0xe9Fve9Fve9GapdbaqaaeGacaGaaiaabeqaamqadiabaaGcbaGaeuiLdqKaemOBa42aa0baaSqaaiabbMgaPjabb6gaUjabbsgaKbqaaiabdofatjabdkfasbaakiabggMi6kabd6gaUnaaDaaaleaacqqGWbaCcqqGVbWBcqqGZbWCcqqG0baDaeaacqWGtbWucqWGsbGuaaGccqGHsislcqWGUbGBdaqhaaWcbaGaeeOyaiMaeeyyaeMaee4CamNaeeyzaugabaGaem4uamLaemOuaifaaOGaeiilaWcaaa@5CD4@

were calculated as well as the changes during normal activity,

ΔnspontSR≡nbaseSR−nctrlSR.
 MathType@MTEF@5@5@+=feaafiart1ev1aaatCvAUfKttLearuWrP9MDH5MBPbIqV92AaeXatLxBI9gBamXvP5wqSXMqHnxAJn0BKvguHDwzZbqegyvzYrwyUfgarqqtubsr4rNCHbGeaGqiA8vkIkVAFgIELiFeLkFeLk=iY=Hhbbf9v8qqaqFr0xc9pk0xbba9q8WqFfeaY=biLkVcLq=JHqVepeea0=as0db9vqpepesP0xe9Fve9Fve9GapdbaqaaeGacaGaaiaabeqaamqadiabaaGcbaGaeuiLdqKaemOBa42aa0baaSqaaiabbohaZjabbchaWjabb+gaVjabb6gaUjabbsha0bqaaiabdofatjabdkfasbaakiabggMi6kabd6gaUnaaDaaaleaacqqGIbGycqqGHbqycqqGZbWCcqqGLbqzaeaacqWGtbWucqWGsbGuaaGccqGHsislcqWGUbGBdaqhaaWcbaGaee4yamMaeeiDaqNaeeOCaiNaeeiBaWgabaGaem4uamLaemOuaifaaOGaeiOla4caaa@5FB6@

**Figure 6:**
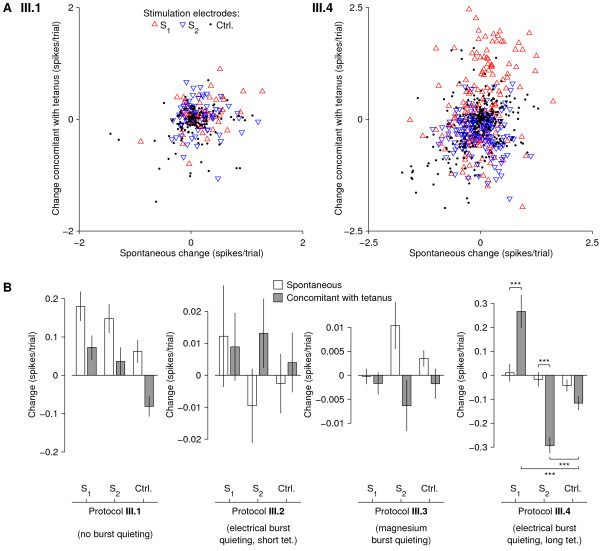
**Results of Series III: Changes induced in specific responses by paired-pulse tetanization**. ** A **Changes induced by tetanization using protocols **III.1 **(*left*) and **III.4 **(*right*) in responses to electrodes *S*_1_, *S*_2 _and the control electrodes, compared to spontaneous drift. Plot symbols indicate stimulation electrode; key applies to both panels. Each point corresponds to one recording electrode in one of 8 experiments. All responses are averaged over 120 presentations of the stimulus. In protocol **III.4 **only, responses to electrodes *S*_1_, *S*_2 _were significantly affected by tetanization. **B **Summary of all experiments. Shown are mean and SEM across all electrode pairs in all experiments for a given protocol (*N *= 67, 67, 142, and 167 for protocols **III.1, III.2, III.3**, and **III.4**). Large bars for spontaneous changes in the panel for protocol **III.1 **indicate ongoing drift. Asterisks above bars for protocol **III.4 **mark significant differences between changes concomitant with tetanization and spontaneous changes: *p *< 0.001 (***), paired t-test. Asterisks below bars indicate significant differences between changes in responses to tetanized electrodes and control electrodes: *p *< 0.001 (***), unpaired t-test.

The results of these calculations for all experiments of protocols **III.1 **and **III.4 **are shown in Figure 6A. It is seen that in protocol **III.4**, but not in protocol **III.1**, responses to electrode *S*_1 _(the electrode that led during the tetani) appear to have been slightly potentiated. At the same time, responses to electrode *S*_2 _(the electrode that followed 5 or 10 ms later) appear to have been slightly depotentiated.

To quantify these observations, we calculated the averages of ΔnindSR
 MathType@MTEF@5@5@+=feaafiart1ev1aaatCvAUfKttLearuWrP9MDH5MBPbIqV92AaeXatLxBI9gBamXvP5wqSXMqHnxAJn0BKvguHDwzZbqegyvzYrwyUfgarqqtubsr4rNCHbGeaGqiA8vkIkVAFgIELiFeLkFeLk=iY=Hhbbf9v8qqaqFr0xc9pk0xbba9q8WqFfeaY=biLkVcLq=JHqVepeea0=as0db9vqpepesP0xe9Fve9Fve9GapdbaqaaeGacaGaaiaabeqaamqadiabaaGcbaGaeuiLdqKaemOBa42aa0baaSqaaiabbMgaPjabb6gaUjabbsgaKbqaaiabdofatjabdkfasbaaaaa@464A@ and ΔnspontSR
 MathType@MTEF@5@5@+=feaafiart1ev1aaatCvAUfKttLearuWrP9MDH5MBPbIqV92AaeXatLxBI9gBamXvP5wqSXMqHnxAJn0BKvguHDwzZbqegyvzYrwyUfgarqqtubsr4rNCHbGeaGqiA8vkIkVAFgIELiFeLkFeLk=iY=Hhbbf9v8qqaqFr0xc9pk0xbba9q8WqFfeaY=biLkVcLq=JHqVepeea0=as0db9vqpepesP0xe9Fve9Fve9GapdbaqaaeGacaGaaiaabeqaamqadiabaaGcbaGaeuiLdqKaemOBa42aa0baaSqaaiabbohaZjabbchaWjabb+gaVjabb6gaUjabbsha0bqaaiabdofatjabdkfasbaaaaa@494A@ across all chosen recording electrodes in all experiments, separately for *S *= *S*_1_, for *S *= *S*_2_, and for *S *= (each of the control electrodes). We found that in protocol **III.4**, responses to electrode *S*_1 _were indeed significantly potentiated by tetanization compared to responses to control electrodes (*p *< 0.001, *N *= 167 electrode pairs). At the same time, responses to electrode *S*_2 _were depotentiated (also *p *< 0.001). Importantly, these changes were significantly greater than spontaneously occurring changes, i.e. differences between control and baseline periods (*p *< 0.001).

In protocol **III.1**, statistically significant differences were also seen between tetanized and control electrodes (*p *< 0.05), but here the changes concomitant with tetanization were not larger than spontaneous changes, so this was not considered an important result. No significant effects were seen in protocols **III.2 **and **III.3.**

## Discussion

Out of all the protocols we tried, only one resulted in statistically significant experimentally induced plasticity. This was surprising, since most of the protocols that did not work were closely related to protocols reported to cause plasticity in dissociated cultures.

### One successful protocol with burst quieting in Series III

In our only successful protocol, **III.4**, stimulation consisted of paired pulses to two electrodes *S*_1 _and *S*_2_. In posttetanus probing, responses to electrode *S*_1 _– which was always stimulated 10 ms before *S*_2 _during tetanization – systematically increased, while responses to electrode *S*_2 _systematically decreased. These changes were significantly larger than spontaneous changes, and also than changes in responses to control electrodes. It is attractive to hypothesize that this effect can be understood to be a consequence of spike timing dependent plasticity (STDP): if *S*_1 _and *S*_2 _converge on a common target cell *R*, and *S*_2 _caused *R *to spike during tetanization, the synapse from *S*_1 _onto *R *would be strengthened, because it was repeatedly activated shortly before the spike. Conversely, if *S*_1 _caused *R *to spike, the synapse from *S*_2 _onto *R *would be weakened, because it was repeatedly activated shortly after the spike.

The fact that in this protocol distributed electrical stimulation was used to quiet bursting supports our earlier hypothesis [29] that spontaneous bursts interfere with plasticity, and thus that controlling bursts is key to controlling plasticity. Protocol **III.2 **used similar burst quieting, yet did not show plasticity greater than pre-tetanus drift. The tetanic stimulation of **III.4 **was longer than that of **III.2**. This suggests that detectable plasticity is evoked only by controlling bursts *and *delivering a sufficiently strong tetanus.

### No successful protocols in Series I

None of the protocols in Series I produced significant stimulus-induced plasticity, in spite of the fact that the induction sequences evoked many culture-wide bursts – whether an induction sequence evoked bursts was a determining factor for whether that sequence could induce plasticity in the experiments of [24]. By elevating slightly the concentration of Mg^2+ ^in the medium (from 0.8 mM to 1.8 or 2.8 mM Mg^2+^), we could (transiently) reduce the intensity of spontaneous bursting. Since bursts could still be evoked by stimulation, we hoped that under these conditions mild potentiation, normally masked by high burst rates, might be revealed. This turned out not to be the case. In summary, our induction sequences failed to have an effect on subsequent spontaneous bursting, by the metrics used here.

### No successful protocols in Series II

In Series II, stimulation-site-specific differences between pre- and posttetanic probing were observed in several protocols. Indeed, our Figure 4A bears a striking resemblance to Figure 2 in [26]. However, our observed stimulation-site-specific differences could not be attributed to the tetanization, since stimulation-site-specific differences of equal magnitude were seen between recording periods without tetanization between them. Instead, we found that the stochastic nature of the occurrence of evoked bursts was responsible for these differences: the occurrence or non-occurrence of a single burst after any one particular stimulus could give rise to differences in spike counts sufficient to explain the apparent stimulation-site-specific potentiation or depotentiation. Whether this non-plastic stochastic effect played a role in the effects reported in [26] can not be ascertained, since in that report the pretetanic period was not broken up into a control and a baseline period, and so no tests were performed to see whether differences in maps could occur without tetanization. Even so, it is, unlikely that it was solely responsible, since in [26] the observed changes depended on the correlation between the responses to test stimuli and the responses to stimulation of the tetanized electrode, while we saw no such dependence.

## Conclusion

Why did our rigorously controlled experiments fail to reveal previously reported plasticity effects (see Table 1)? One possibility is that subtle differences in culturing conditions (see Table 5) made our cultures less amenable to inducing plasticity. Certainly, substantial differences existed even between different plating batches created within our lab [30]. However, our results are based on 112 experiments on 18 cultures from 4 dissections, so it is unlikely that we simply had an unlucky pick. Another is that previous experiments insufficiently controlled for drift; in most protocols, we found that changes between baseline recordings and post – induction sequence were significantly larger than trial-to-trial variability (suggesting that plasticity occurred), but comparison with control intervals always revealed that changes of similar magnitude had also occurred spontaneously (i.e., between control and baseline periods, in the absence of induction sequences). Thus, the causation of changes could not be attributed to the induction sequence.

**Table 5:** Overview of culturing and recording conditions used by other researchers. The following is a synopsis of conditions reported in the *Methods *section of the papers listed in Table 1.

						Ion concentrations (mM)						
Ref.	T (°C)	Perfusion?	pH	Age at time of Expts.	Culture Medium	Ca^2+^	Mg^2+^	K^+^	Osmolarity (mOsm)	Plating Density (/mm^2^)	Observed Density (/mm^2^)	Type of Stim.
[24]	20	Y	7.2	1–6 w	DMEM-5/5	1	0–2	2.8	330–334	10, 000	-	V
[25]	-	-	7.2	1–5 w	DMEM-5/5	1	1–10	2.8	336–354	-	-	V
[26]	-	-	-	30–50 d	DMEM-5/5	-	-	-	-	-	120	V
[27]	-	Y	7.2	40–50 d	DMEM-5/5	2	1	2.8	334	-	175	V
[3]	37	-	-	3 w	MEM-5HS	-	-	-	-	-	300	I
[5]	37	-	-	3 w–3 m	MEM-5FBS	1.8	0.8	5.4	330	8000	-	V
*This paper*	35	N	7.2	10–32 d	DMEM-10HS	1.8	0.8	5.4	330	2700	2500	V

Notes: Parameters not explicitly mentioned in a paper are represented as '-' in the table. DMEM-5/5 refers to Dulbecco's Modified Eagle's Medium with 5% Fetal Bovine Serum and 5% Horse Serum. MEM-5HS is probably Modified Eagle's Medium with 5% Horse Serum, but the abbreviation 'MEM' is not explained in [3]. MEM-5FBS is Modified Eagle's Medium with 5% Fetal Bovine Serum, and DMEM-10HS is Dulbecco's Modified Eagle's Medium with 10% Horse Serum. "Plating Density" is the cell density at time of plating reported in the paper, while "Observed Density" is the density we counted in photographs in the paper or a reference in its Methods section to an earlier paper by the same authors. "Type of Stim." indicated whether the stimuli used were defined in terms of voltage (V) or of current (I).

We are left to conclude that inducing plasticity by extracellular electrical stimulation in dense dissociated cortical cultures is not as straightforward as it is in brain slices [20]. There are several possible explanations why this might be so. It could be that cultures burst too much, causing plastic change to be reversed quickly [36]. Alternatively, it could be that synapses tend to be already saturated in culture, perhaps as a result of the thousands of bursts that the cultures had experienced before these experiments took place [37]. A third possible explanation is that neurons in culture lack critical neuromodulatory input during development. We are presently pursuing the possibility, suggested by the main positive result in this paper, that burst quieting by distributed electrical stimulation [29] may reveal plasticity effects that otherwise cannot be observed – either because they are buried in drift, or because they do not last long enough [38], or because they do not occur at all.

We hope that this detailed presentation of (mostly) negative results will help make it easier to discuss in print how difficult it is to induce plasticity in cultured networks using extracellular electrodes. Such discussions may reveal the crucial variables that reproducibly permit induction of plasticity with MEAs. That will allow this accessible model system to be more relevant to in vivo mechanisms of learning and memory.

## Methods

### Cell culture

Cultures were prepared and maintained as before [30]. Briefly, cortical cells – neurons and glia in natural proportions – were obtained from E18 rat embryos, and 50,000 cells in a 20 *μ*L drop were plated over the center of MEAs (MultiChannel Systems, Reutlingen, Germany). This resulted in monolayer cultures of 5 mm diameter – three times larger than the diameter of the electrode array – with a density of about 2,500 cells/mm^2 ^after one day *in vitro *(div). Cultures were sealed with Teflon membranes [6], and maintained at 35°C, 5% CO_2_, and 9% O_2_, in a serum-containing DMEM-based medium adapted from [26]. Experiments were performed after 10–32 div, in the same incubator in which cultures were maintained.

### Delivery of chemicals

To deliver chemicals (obtained from Sigma) to a culture, we used a stock solution of at least 20× concentration in DMEM (Irvine Scientific) and added drops of at most 50 *μ*L directly to the 1 mL of culture medium present in the MEA dish, to avoid any transients due to medium exchange. To ensure equal distribution of the applied substance through the entire medium, 0.5 mL of medium was taken out near the spot where the drop was added, and gently returned on the opposite side of the dish. This technique was verified by applying a small drop of strong acid to a culture dish with pH-indicator medium. After mixing in the manner described, the color of the medium became a uniform orange.

### Electrical recording and stimulation

Multielectrode arrays with 59 electrodes were used for both recording and stimulation. Recorded signals were bandpass filtered (10 Hz–5 kHz), amplified, and digitized using an MEA1060 preamplifier and MC_Card data acquisition board (both from Multichannel Systems). Software signal processing, including artifact suppression and spike detection, was performed online using our open-source software MeaBench [39]. Further analysis was performed using custom Matlab code (MathWorks, Natick, MA, USA).

Voltage-controlled stimuli were delivered using our custom multi-site stimulator [40]. All pulses were biphasic, 400 *μ*s per phase, positive first, as optimized in [33]. Care was taken to limit voltages to less than 1 V, to avoid electrochemically damaging cells or electrodes. All 59 electrodes in the array could be used for stimulation, but due to a broken wire in one pre-amplifier channel, only 58 could be used for recording. Stimulation artifacts were commonly observed on several electrodes, but could be adequately suppressed in software using SALPA [41], and spike detection was possible on all but the stimulated electrode within 2 ms post-stimulus. (The stimulated electrode could again be used for recording 50 ms later.)

### Electrical burst quieting

In several protocols, culture-wide spontaneous bursting was suppressed using distributed electrical stimulation [29]. Twenty to forty electrodes that evoked responses in pre-experimental probing were arbitrarily selected, and stimuli were delivered to each of them in cyclic order, with an inter-stimulus interval of 20 ms, so each electrode was stimulated 1.25–2.5 times per second. This resulted in a complete but reversible cessation of spontaneous bursting.

### Choice of electrodes

Effective stimulation requires good contact between the stimulated electrode and the culture: if a pulse cannot elicit a response, it is unlikely that it will induce plasticity. Therefore, all experiments began with probing each electrode in the array with pulses of several voltages: each electrode was stimulated 27 times at 100–900 mV; the sequence was fully randomized; inter-stimulus intervals were 0.3 s. Electrodes with strong contact with the culture should elicit a graded response: with increasing voltage, more and more cells should be recruited [33]. For induction of plasticity, electrodes were chosen that (1) clearly showed this effect, and (2) which could be used to increase the array-wide spike detection rate (ASDR) to at least twice the baseline level, using pulses no larger than 900 mV. In all cultures, many electrodes fulfilled these requirements (10–50). When multiple experiments were performed on a single culture, we selected electrodes from different regions of the array for each new experiment, to maximize independence between experiments.

### Reducing probe pulse amplitudes in order to attempt to avoid evoking bursts

In protocol **II.4 **we attempted to reduce the likelihood that probe pulses evoked bursts, as follows. Before each experiment, each electrode in the array was probed at 19 voltages between 40 and 580 mV. Stimuli were presented in random order at 3 s intervals. For each electrode, the lowest voltage that ever evoked a burst was determined, and the amplitude of test pulses used in the main experiment was set to two thirds of this voltage. Unfortunately, it transpired that in the absence of stronger stimuli many of these relatively weak stimuli could still evoke bursts. This rendered the reduction of stimulation voltages futile (though harmless). Importantly, it does not imply that plasticity was induced. Instead, it can likely be understood as follows. If stimuli are presented at a rate faster than the spontaneous burst rate of the culture, only some of the stimuli will trigger bursts, due to burst refractoriness. If the stimulation is a mixture of strong and weak pulses, most bursts will be entrained to the strong pulses, because a strong pulse will more easily evoke a burst during the relative refractory period following a previous burst. Thus, weak pulses never have a chance to evoke bursts. When there are no strong pulses, the network is never made refractory to the weaker pulses, which may thus get their chance to evoke bursts. This mechanism makes the entrainment of bursts context-dependent, but does not imply plasticity in the sense used by most researchers of long-term synaptic plasticity.

### Statistics

For all comparisons between spontaneous and putatively induced changes, data were pooled from all experiments in which a given protocol was used, and paired t-tests were used to assess significance. In Series III, differences between effects on responses to *S*_1_*, S*_2_, and the control electrodes were tested using unpaired t-tests. Note that in Figure 3A and 3B error bars were based on the assumption that burst generation is approximately Poisson in nature. In practice, bursting is likely more regular, so our error bars are probably overestimated. Importantly, for the tests in Figure 3C and 3D, the nature of the burst generation process is not important, so whether or not it is Poisson does not affect our final conclusions.

## Abbreviations

ASDR = array-wide spike detection rate

base = baseline

ctrl = control

div = days *in vitro*

ind = induction

MEA = multielectrode array

rec = recording-site specific

post = post-induction

SALPA = Suppression of Artifacts by Local Polynomial Approximation[41]

spont = spontaneous

stim = stimulation-site specific

## Competing interests

The author(s) declare that they have no competing interests.

## Authors' contributions

DAW collected all data, performed the analysis, and prepared text and figures for the manuscript. JP and SMP contributed to the design of the study and to the preparation of the manuscript.
