# Correction: searching for plasticity in dissociated cortical cultures on multi-electrode arrays

**DOI:** 10.1186/1477-5751-6-3

**Published:** 2007-02-08

**Authors:** Daniel A Wagenaar, Jerome Pine, Steve M Potter

**Affiliations:** 1 Division of Biological Sciences, Neurobiology Section, University of California at San Diego, 9500 Gilman Drive, La Jolla, CA 92093, USA; 2 Department of Physics, California Institute of Technology, Caltech 256-48, Pasadena, CA 91125, USA; 3 Coulter Department of Biomedical Engineering, Georgia Institute of Technology and Emory University, 313 Ferst Drive, Atlanta, GA 30332-0535, USA

After publication of this article [1], the authors became aware that the numerical codes for two of the datasets, protocols **III.3 **and **III.4**, were inadvertently confused during the writing of the paper. Accordingly, the following corrigenda apply:

• *Abstract*, last line: For 'distributed electrical stimulation,' read 'elevated extracellular magnesium concentrations.'

• *Background*, last paragraph before 'Results': For 'distributed multi-site stimuli [29],' read 'elevated extracellular magnesium concentrations.'

• *Results*, third to last paragraph (after the equation 'ΔnspontSR≡nbaseSR−nctrlSR
 MathType@MTEF@5@5@+=feaafiart1ev1aaatCvAUfKttLearuWrP9MDH5MBPbIqV92AaeXatLxBI9gBaebbnrfifHhDYfgasaacH8akY=wiFfYdH8Gipec8Eeeu0xXdbba9frFj0=OqFfea0dXdd9vqai=hGuQ8kuc9pgc9s8qqaq=dirpe0xb9q8qiLsFr0=vr0=vr0dc8meaabaqaciaacaGaaeqabaqabeGadaaakeaacqqHuoarcqWGUbGBdaqhaaWcbaGaee4CamNaeeiCaaNaee4Ba8MaeeOBa4MaeeiDaqhabaGaem4uamLaemOuaifaaOGaeyyyIORaemOBa42aa0baaSqaaiabbkgaIjabbggaHjabbohaZjabbwgaLbqaaiabdofatjabdkfasbaakiabgkHiTiabd6gaUnaaDaaaleaacqqGJbWycqqG0baDcqqGYbGCcqqGSbaBaeaacqWGtbWucqWGsbGuaaaaaa@4E89@'): For '**III.4**,' read '**III.3**' (two instances), and for '5 or 10 ms later', read '5 ms later.'

• *Results*, second to last paragraph: For '**III.4**,' read '**III.3**.'

• *Results*, final paragraph: For '**III.3**,' read '**III.4**.'

• *Figure 6A*, right panel: For '**III.4**,' read '**III.3**.'

• *Figure 6B*, two rightmost panels: For '**III.3 **(magnesium burst quieting),' read '**III.4 **(electrical burst quieting, long tet.)' and vice versa.

• *Figure 6*, caption: For '**III.4**,' read '**III.3**' and vice versa (several instances). In particular: '*N *= 67, 67, 167, 142 for protocols **III.1**, **III.2**, **III.3**, and **III.4**.'

• *Discussion*, first paragraph following the caption *One successful protocol with burst quieting in Series III: *For '**III.4**,' read '**III.3**.' For '10 ms,' read '5 ms.'

• *Discussion*, following paragraph: This paragraph ('The fact that ... tetanus') should be dropped entirely.

A version of the article with the above corrections marked is available from the authors' website [2].
